# Development and evaluation of the Norwegian Fatigue Characteristics and Interference Measure (FCIM) for stroke survivors: cognitive interviews and Rasch analysis

**DOI:** 10.1007/s11136-023-03477-z

**Published:** 2023-07-19

**Authors:** Ingrid Johansen Skogestad, Anders Kottorp, Petra Larsson, Therese Marie Moen, Caryl L. Gay, Christine Råheim Borge, Anners Lerdal

**Affiliations:** 1 Medical Department, Lovisenberg Diaconal Hospital, Oslo, Norway; 2 Department of Public Health Science, Faculty of Medicine, Institute of Health and Society, University of Oslo, Oslo, Norway; 3 Faculty of Health and Society, Malmö University, Malmö, Sweden; 4 Surgical Department, Lovisenberg Diaconal Hospital, Oslo, Norway; 5 Department of Interdisciplinary Health Sciences, Faculty of Medicine, Institute of Health and Society, University of Oslo, Oslo, Norway; 6 Research Department, Lovisenberg Diaconal Hospital, Oslo, Norway; 7 Department of Family Health Care Nursing, University of California, San Francisco, USA

**Keywords:** Fatigue, Stroke, Patient-reported outcome measure (PROM), Measurement properties, Cognitive interviews, Rasch analysis

## Abstract

**Purpose:**

There is need for a comprehensive measure of post-stroke fatigue with sound measurement properties. This study aimed to develop the Norwegian Fatigue Characteristics and Interference Measure (FCIM) and assess its content validity, structural validity, and internal consistency.

**Method:**

This study consisted of three steps: (1) an expert panel developed version 1.0 of the Norwegian FCIM, (2) its content validity was assessed in cognitive interviews with stroke patients (*N* = 15), (3) a convenience sample of stroke patients (*N* = 169) completed an online questionnaire with the FCIM, Fatigue Severity Scale, and sociodemographic information; validity and reliability were assessed using Rasch analysis.

**Results:**

FCIM version 1.0 included a 10-item characteristics subscale, a 20-item interference subscale, and two pre-stroke fatigue items. The cognitive interviews revealed content validity issues, resulting in two interference items being removed and five items being flagged but retained for Rasch analysis (version 2.0). Rasch analysis led to removal of four items from the characteristics subscale and six more from the interference subscale. The final six-item characteristics subscale and 12-item interference subscale (version 3.0) both showed adequate fit to the Rasch model with indications of unidimensionality and local independence. The interference subscale had a high person separation index. No significant differential item function (DIF) was found in relation to gender, but one item demonstrated DIF in relation to age.

**Conclusion:**

The cognitive interviews and Rasch analysis demonstrated that the Norwegian version of the FCIM has high content validity, structural validity, and internal consistency. Future research should assess its construct validity, reliability, and responsiveness.

**Supplementary Information:**

The online version contains supplementary material available at 10.1007/s11136-023-03477-z.

## Introduction

Fatigue is a common and debilitating symptom for stroke survivors, potentially affecting every part of their daily activities and quality of life [1]. Stroke clinicians are currently advised to systematically assess fatigue in all patients [2, 3]. However, there is insufficient evidence to support the use of any routine treatment or prevention strategies [4, 5], and the pathophysiology of post-stroke fatigue (PSF) is largely unknown [1]. A critical barrier in PSF research is the lack of a patient-reported outcome measure (PROM) for fatigue with sound psychometric properties [6, 7].

There is growing recognition that content validity is the most important measurement property of a PROM [8]. Content validity can be defined as “the degree to which the content of a measurement instrument is an adequate reflection of the construct to be measured” [9]. It is advised that content validity should be demonstrated before evaluating other psychometric properties [10, 11]. However, establishing content validity in PROMs that assess unobservable constructs such as fatigue is challenging and requires several steps involving qualitative methods [9]. In a prior study [12], we explored the experience of fatigue in a qualitative study with stroke survivors and health professionals. These findings resulted in the development of a conceptual framework that outlined PSF as a multidimensional phenomenon. Two important dimensions were fatigue characteristics (e.g., intensity, timing) and fatigue interference (i.e., emotional, cognitive, activity, and social impacts of fatigue). A clear definition of the construct to be measured is a prerequisite for item development [8]. Despite this, recent fatigue measures lack a clear definition, measure various aspects, and lack high-quality evidence of content validity [6].

In stroke research, the Fatigue Severity Scale (FSS) is the most-used PROM for fatigue. However, the FSS lacks evidence of content validity and evidence of its psychometric properties is limited, particularly among people with stroke [6, 13, 14]. For example, the FSS does not assess important features such as mental versus physical fatigue or diurnal variations [6, 15]. Although other, more complex PROMs for fatigue exist, a recent review found no multidimensional fatigue questionnaires had been adequately validated in people with stroke [16]. With the growing recognition of PSF as a unique clinical entity and the increasing use of PROMs as endpoints in clinical studies, there is a clear need to develop a PSF-specific PROM with robust measurement properties that capture multidimensional features of PSF.

After establishing content validity, guidelines also recommend extensive field testing to obtain insight into the structural validity of the data [9, 11, 17]. Structural validity can be defined as “the degree to which the scores of a measurement instrument are an adequate reflection of the dimensionality of the construct to be measured” [18]. Rasch analysis is a restrictive model that builds a linear interval measure invariant across test-takers and is suitable for item reduction and to examine structural validity and item characteristics in detail [9, 19]. Further, Rasch analysis can obtain stable parameter estimates with smaller sample sizes than 2-parameter item response theory models [20]. The fatigue instruments currently used in stroke populations have limited evidence of their content validity. Studies have often moved straight to assessing other types of validity, reliability, and responsiveness [13, 16, 21]. This approach could introduce several potential biases in these instruments’ scores [8]. To move forward in PSF research, there is need for a fatigue PROM designed for and validated in the stroke population and that has been developed following advanced PROM guidelines [16]. The aim of this study was, therefore, to develop such a measure and evaluate its content validity, structural validity, and internal consistency.

## Method

### Design

**Fig. 1:**
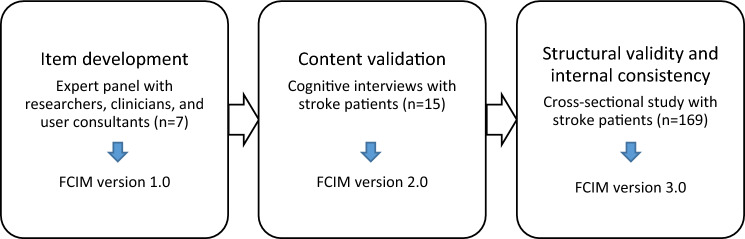
The three iterative steps in the development of the Norwegian FCIM

This study had a mixed-methods design involving three iterative steps (Fig. 1), as described by de Vet et al. [9]. An expert panel developed the instrument’s initial items, cognitive interviews were conducted to evaluate content validity, and an online questionnaire was used to collect data for a Rasch analysis guiding item reduction and evaluating structural validity and internal consistency. Reporting of the item development and cognitive interviews follows the consolidated criteria for reporting qualitative research [22], and reporting of the Rasch analysis follows the Rasch reporting guideline for rehabilitation research [23].

### Item development

We first established an expert panel, using convenience sampling to include a balanced group with diverse backgrounds. The expert panel consisted of three stroke researchers and clinicians from Lovisenberg Diaconal Hospital (LDS), two PROM researchers (one from LDS and one external expert on Rasch analysis), and two stroke patients with fatigue serving as user consultants. The first author (IJS), who is a nurse and current PhD student, led six meetings (four live at LDS and two on Zoom) each lasting 1–4 h (with breaks). Between meetings, all group members read and commented on written versions of the instrument. The expert panel developed the items and response categories for the fatigue instrument based on our conceptual framework of fatigue derived from a previously published qualitative study. In that study, we defined PSF as “an experience of mental, physical or general feeling of exhaustion and tiredness, with a discrepancy between the level of activity and the level of fatigue” [12]. The expert panel developed the initial instrument informed by PROM development guidelines [8, 24–27]. The aim was to develop an instrument that could measure characteristics and interference (in two subscales), have the potential to measure improvement (for use in intervention studies), assess the level and duration of fatigue experienced prior to stroke (i.e., pre-stroke fatigue) and be relatively short (feasible). The expert panel developed version 1.0 of the instrument, named the Fatigue Characteristics and Interference Measure (FCIM), through regular meetings from January through June 2020. We also consulted a speech therapist for advice on adapting the instrument for people with aphasia and/or reading difficulties. Finally, to ensure item clarity and test interview technique, we performed two pilot interviews with the user consultants.

### Cognitive interviews—developing evidence of content validity

Next, we conducted cognitive interviews with stroke patients. The aim was to establish the content validity of FCIM version 1.0 by evaluating comprehensibility, retrieval, judgment and communication, comprehensiveness, and relevance [26, 28]. A stroke user organization in Norway invited members to participate via text messages, e-mail, and its Facebook page. Inclusion criteria were prior stroke during the last 2 years, over 18 years, and living within driving distance from Oslo. 15 stroke survivors were purposively sampled to ensure variability in age, gender, fatigue severity, and time since stroke diagnosis. Face-to-face individual interviews were conducted during August and September 2020. Eight interviews were conducted in participants’ homes, and seven at LDS in Oslo. Participants completed FCIM version 1.0 as part of the interview, and a questionnaire including a 7-item version of the FSS (FSS7) [14] and information about their sociodemographic characteristics and relevant medical history.

Cognitive interviews were conducted with the Three-Step Test-Interview (TSTI) technique [29] and followed a semi-structured interview guide (Online Resource 1). TSTI was developed as an aid to identify problems in newly developed instruments. It consists of the following three steps:

1. *Observing* response behavior and concurrent *thinking aloud*. The interviewer takes notes and observes behavior such as hesitation and correction of response category. The participants are also instructed to think aloud and verbalize their thoughts when filling out the instrument. The aim is to make the participants’ immediate thoughts about the instrument observable for the interviewer.
2. *Follow-up probing* considering the behavior or expressed thoughts collected in step 1, where the aim is to clarify and complete only the primary data previously collected.
3. *Debriefing* aimed at eliciting experiences and opinions, such as potential problems, possible improvements, and the instrument’s completeness.

Audio recordings and field notes were taken during the interviews. Recordings were transcribed verbatim and subject to a deductive content analysis facilitated by NVivo 12 [30]. Each item was analyzed separately, and we used a categorization matrix based on Tourangeau’s four-stage cognitive model [28], which includes comprehension, retrieval, judgment, and response. Finally, we assessed the completeness of the instrument as a whole. After 10 interviews, some changes were made to the instrument, and then again after completing all 15 interviews, resulting in FCIM version 2.0.

### Rasch analysis—developing evidence of structural validity and internal consistency

We conducted a cross-sectional study with a convenience sample of stroke patients (*N* = 169). Participants were recruited through the website of a stroke user organization. Inclusion criteria were adults with a self-reported stroke diagnosis who could read Norwegian. Data were collected between January and March 2021. Participants responded to an online questionnaire including sociodemographic information, relevant medical history, FSS7, and FCIM version 2.0.

FCIM version 2.0 was analyzed using a Rasch model which calculates the probability of a specified response for both persons and items along the same linear scale (representing the latent trait). This enables transformation of ordinal raw scores into an interval-level variable (called logits). Winsteps (version 5.2.0.0), R (version 4.1.2), and SPSS (version 28.0.0.0) were used to conduct statistical analyses and generate graphs. Since the FCIM is a new instrument and includes items with different response categories, we applied a Partial Credit Model (PCM) which make no assumptions about the equidistance between thresholds across items [31]. Then we assessed rating scale functioning according to Linacre’s guidelines to determine whether the scale was suitable for Rasch analysis [32] (Table 1). The primary focus in the Rasch analysis was to address two main aims: item reduction and evaluation of FCIM’s structural validity and internal consistency. This process involves repeated analyses (iterations) of each subscale in FCIM. Following each iteration, the measurement properties were evaluated and items not meeting pre-established criteria were removed. Iteration of analysis were repeated until satisfactory results, as outlined in Table 1.

**Table 1:** Overview of measurement properties and pre-set criteria assessed for and through Rasch analysis

Measurement properties	Aim	Criteria
Rating scale functioning	Assess item responses and their fit to the Rasch model assumptions	Assessment of distribution At least 10 observations of each category Categories advance monotonically Outfit MNSQ < 2.0 Step calibrations (Andrich Thresholds) advance between 1 and 5 logits
Item goodness of fit	Assess individual items’ fit to the Rasch model	Infit MNSQ between 0.7 and 1.3 [42]
Structural validity	Principal components analysis (PCA) of the residuals to assess unidimensionality and local independence	Unidimensionality is supported when: raw variance explained by the measure is > 50% of the total variance, and that the variance explained by the first principal component is small, i.e., < 2 eigenvalues [35] Yen’s Q3 correlations between residuals < 0.3 [36, 43]
Internal consistency	Targeting of persons with mean and S.E of θ (theta)	Report direction and distance from mean item measure at 0 [43]
Internal consistency	Assess person separation index and reliability Assess item separation index and reliability	Person separation > 2 Person reliability ≥ 0.8 Item separation > 3 Item reliability > 0.9 [35]
Internal consistency	Assess consistency in correlations between items	KR-20 ≥ 0.70 for each unidimensional scale or subscale [43]
Person goodness of fit	Detect improbable item-score patterns	Infit MNSQ ≤ 1.4 and/or associated z-value < 2 [14]
Uniform differential item function (DIF)	Assessed across gender and three age groups (24–44, 45–60, 61–83) selected based on previous known curvilinear relationship with PSF and age [38]	Uniform DIF was analyzed using Mantel Chi-Square test for polytomous data with a Bonferroni- adjusted p-value = 0.01 [14, 44]

Rasch analysis uses fit statistics to estimate how well the items’ and persons’ raw data fit the model assumptions [33]. Fit statistics are presented in infit and outfit unstandardized mean square (MNSQ) and standardized fit statistics (z-values). The MNSQ residuals show the degree of randomness and a MNSQ value of 1.0 indicates perfect fit. Values less than 1.0 indicate overfit to the model (i.e., the observations are too predictable), while values greater than 1.0 indicate underfit (i.e., there is more randomness in the data than expected in the Rasch model). Consistent with earlier empirical studies [14], we evaluated standardized infit statistics as they are more sensitive to unexpected item response patterns targeted to the person. This is in contrast to outfit, which is more sensitive to unexpected observations on very easy or hard items, with high outfit MNSQ often resulting from a few random responses by low performers [33, 34].

#### Structural validity

Each subscale’s unidimensionality was assessed separately using Principal Components Analysis (PCA) of the residuals [35]. Although the conceptual framework of fatigue is multidimensional, statistical unidimensionality of each subscale/dimension needs to be ensured. Local dependency between items was evaluated with Yen’s Q3 statistics, which compute raw score residual correlations [36]. Local independence means that the variance left after removing the contribution to the latent trait is only random, normally distributed, noise [35]. If two items are locally dependent, it indicates either that they add to some other dimension, or that they duplicate some feature of each other (called redundancy-dependency) [35].

#### Internal consistency

Targeting of persons was reported with the mean measure score (theta) for persons and mean standard error. Well-targeted measures have similar mean locations for persons and items. A positive mean value for persons indicates higher levels of fatigue compared to the average of the scale (set at 0) and a negative value indicates lower levels of fatigue [37]. We also reported Wright item maps that, on the same scale, display both the individual participants’ ability measures and the individual items’ difficulty calibrations (including the step calibrations [Andrich thresholds]) [35]. Precision was evaluated using the person and item separation index with associated reliability [35]. Consistency in item correlations was assessed with Kuder-Richardson Formula 20 (KR-20).

#### Score-to-measure conversion

Rasch analysis converts the raw scores to interval data (logits). To facilitate clinical interpretation of FCIM, we provide score-to-measure tables for both subscales.

The groups of persons with misfitting MNSQ and z-values were compared to the rest of the sample concerning age, gender, and fatigue (FSS severe fatigue vs. no/mild/moderate fatigue (combined)), using Student’s t test or Fisher’s exact test, as appropriate.

#### Uniform differential item functioning (DIF)

DIF analysis was conducted to investigate whether previously known subgroups (gender and age [1, 38]) had significantly different responses to items despite equal levels of the underlying trait [35].

### Ethical considerations

The study was conducted following the Declaration of Helsinki and was approved by the Regional Medical and Health Ethics Committee of Southeastern Norway (REK) (reference 2017/1741). All participants provided written informed consent.

## Results

### Instrument development by expert panel

First, the expert panel generated 160 items related to our conceptual framework [12]. Then, the panel selected the best-worded and most relevant, comprehensive, and discriminating items [18]. The FCIM is based on a reflective model and was developed after thorough discussions and according to relevant guidelines [8, 26]. Based on the conceptual framework, the instrument was divided into two subscales: Characteristics and Interference. This initial version consisted of our definition of PSF and 32 items with a 7-day recall period. The Characteristics subscale had 10 items with a 5-point Likert scale ranging from “not at all” to “very much.” Three item pairs (fatigued items 1 and 2, mental fatigue items 3 and 4 and physical fatigue items 5 and 6) were designed to be the same question, but with slightly different wording (Table 2). We included all three item pairs to investigate in the next steps which items were preferred by stroke patients. The Interference subscale had 20 items with a 5-point Likert scale ranging from “never” to “all the time.” In addition, we included two pre-stroke fatigue items to help distinguish post-stroke fatigue from pre-existing levels of fatigue. Based on the speech therapist’s input, we used bold font for essential words in each item. This process resulted in FCIM version 1.0.

**Table 2:** Version 2.0 of the Norwegian Fatigue Characteristics and Interference Measure (FCIM)

Version 2.0 of the Norwegian Fatigue Characteristics and Interference Measure (FCIM)^a^		
In this questionnaire, we want to assess post-stroke fatigue. It is very normal to feel tired during periods when you have a lot to do, but being fatigued means that you are more tired than you would expect considering what you have done		
Please choose the answer that best describes how you have been feeling in the past 7 days		
To what degree did you feel	Not at all—a little bit—somewhat—quite a bit—almost always	
1. Fatigued		
2. *Exhausted*		
3. Mentally fatigued		
4. *Tired in your head*		
5. Physically fatigued		
6. *Tired in your body*		
7. Fatigued in the morning		
8. Fatigued around noon		
9. Fatigued in the afternoon		
10. *Fatigued in the evening*		
Challenges due to fatigue	Never – Rarely – Sometimes – Often – All the time	
11. How often were you so fatigued that you had problems **concentrating**?		
12. How often were you so fatigued that you had problems **making decisions**?		
13. How often were you so fatigued that you had problems **following a conversation**?		
14. *How often were you so fatigued that you had problems ****gathering your thoughts****?*		
15. *How often were you so fatigued that you had problems taking a ****bath or shower****?*		
16. *How often were you so fatigued that you had problems getting ****dressed/undressed****?*		
17. How often did you have problems **starting** your tasks/activities because of fatigue?		
18. *How often did you have problems ****completing**** your tasks/activities because of fatigue?*		
19. How often did you have to **give up on** your tasks/activities because of fatigue?		
20. How often have tasks/activities **taken more time** because of fatigue?		
21. How often did you avoid **activities outside your home** because of fatigue?		
22. How often has it been difficult to **plan activities** ahead of time because of fatigue?		
23. How often have you felt fatigued even if you have **not done anything?**		
24. How often did you avoid **physical activity** because of fatigue?		
25. How often did you limit your **social activities** because of fatigue?		
26. How often were you too fatigued to be **together with your family**?		
27. *How often did you avoid ****engaging in hobbies or leisure activities**** because of fatigue?*		
28. *How often did you avoid ****pleasant activities**** because of fatigue?*		
Pre-stroke fatigue		
29. Before you had a stroke, to what degree did you feel fatigued then? Not at all – A little bit – Somewhat – Quite a bit – Almost always		
30. Before you had a stroke, for how long had you felt fatigued? (If item 29 is greater than ‘Not at all’) 1 month or less – 2–6 months – 7–12 months – more than a year		

^a^The FCIM was developed in Norwegian and was translated above by the first author in the interest of this publication only. At the time of publication, the measure has not yet been translated into English according to the current standards for translation of measures for research purposes. This version should not be considered the final version and should not be used

The 10 items in italics (2, 4, 6, 10, 14, 15, 16, 18, 27, and 28) were evaluated in FCIM version 2.0, but were not included in the final 20-item version 3.0

### Content validity testing by cognitive interviews

FCIM version 1.0 was then assessed in 15 cognitive interviews with stroke patients. Characteristics of the participants are presented in Table 3. The interviews lasted between 24 and 92 (mean 53) minutes. Details about the cognitive interviews results are displayed in an item-tracking matrix (Online Resource 2). Preliminary analysis after the first 10 interviews showed difficulties with comprehension of the PSF definition, and minor difficulties with comprehension and judgment in seven items in the Interference subscale. We edited the instrument and presented the updated version in the next five interviews, resulting in improved understanding of these items and the PSF definition. After 15 interviews, we changed the ordering of items in the Interference subscale and removed two items due to comprehension problems and lack of relevance. We also flagged five items (2, 4, 6, 27 and 28) because of similarity, comprehension and judgment issues. Despite potential issues, we decided to temporarily keep the flagged items and investigate their performance in the Rasch analysis. This resulted in FCIM version 2.0 with 10 Characteristics items, 18 Interference items and two pre-stroke fatigue items (Table 2). Except for the five flagged items, we found no significant problems relating to comprehension, judgment, relevance, or completeness of the items.

**Table 3:** Patient characteristics for the cognitive interview and Rasch analysis samples

Patient characteristics	Cognitive interviews (*n* = 15)	Rasch analysis (*n* = 169)
Age, mean (range)	55.5 (40–75)^a^	52.4 (24–83)
24–44	2	35 (20.7)
45–60	7	98 (58.0)
61–83	4	36 (21.3)
Male, *n* (%)	7 (46.7)	66 (39.1)
Education, *n* (%)		
Primary school	2 (13.3)	11 (6.5)
Secondary school	5 (33.3)	62 (36.7)
Higher education < 4 years	4 (26.6)	61 (36.1)
Higher education ≥ 4 years	4 (26.6)	35 (20.7)
Marital status, *n* (%)		
Married	11 (73.3)	95 (56.2)
Unmarried	3 (20.0)	38 (22.5)
Widowed	0	4 (2.4)
Divorced/separated	1 (6.6)	32 (18.9)
Years since stroke, *n* (%)		
1–24 months	13 (86.6)	69 (41)
> 2 years	2 (13.3)	100 (59)
Type of stroke, *n* (%)		
Cerebral infarction	13 (86.6)	126 (75)
Hemorrhage	2 (13.3)	34 (20)
Unknown/other	0	9 (5)
Work status, *n* (%)		
Working (full or part time)	5 (33.3)	60 (35.5)
Stroke-related speech disorder, *n* (%)	Not collected	55 (32.5)
Weekly rehabilitation with speech therapist, *n* (%)	1 (6.7)	12 (7.1)
Fatigue Severity Scale (FSS7) total score^b^, *n* (%)		
No/mild fatigue (1–3.9)	3 (20.0)	21 (12.4)
Moderate fatigue (4–4.9)	5 (33.3)	23 (13.6)
Severe fatigue (5–7)	7 (46.7)	125 (74.0)

All data are self-reported

^a^Age is missing for two cognitive interview participants

^b^The FSS total score is calculated as the mean of all item scores, can range from 1 to 7 and can be divided into the categories above [45]

### Evaluating structural validity and internal consistency with Rasch analysis

FCIM version 2.0 was further evaluated in a sample of 169 patients with stroke who responded to an online questionnaire (Table 3). There were no missing data. First, we evaluated the functioning of both subscales against Linacre’s guidelines (Table 1) [32], and both subscales fulfilled the criteria of having unimodal distribution with peaks in the center, more than 10 observations in each category, outfit MNSQ < 2.0, and step calibrations (Andrich thresholds) that advanced monotonically between 1 and 5 logits (Online resource 3 and 4).

#### Characteristics subscale

**Fig. 2:**
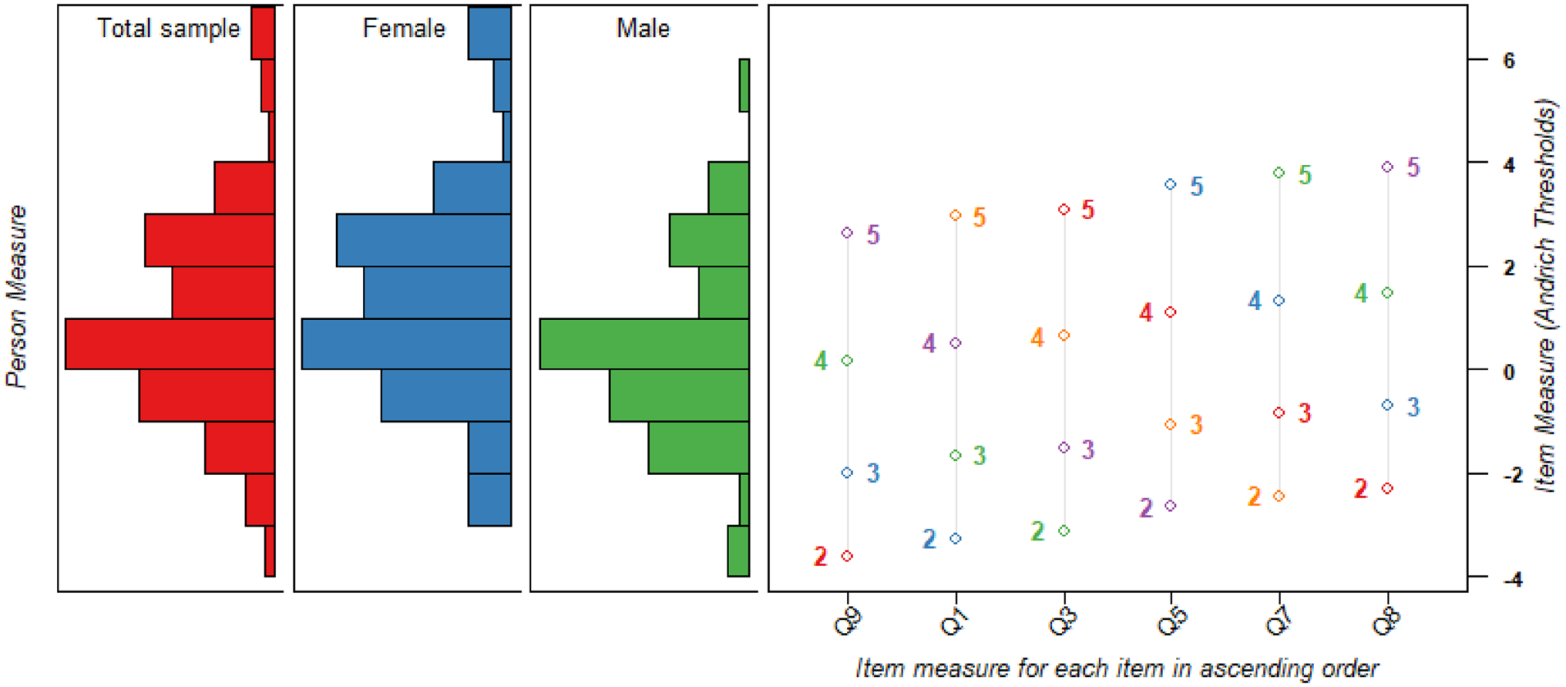
Wright map displaying the 6-item Characteristics subscale. The left side displays individual participants’ ability measures (based on their mean logits), presented both as a total sample, and separately for females and males. The right side displays the individual items’ difficulty calibrations, including the difficulty of each step calibration (Andrich thresholds). Both the participants’ ability measures and the individual item difficulty calibrations are spaced along the common vertical axis with the logits presented on the right side [35]

The first iteration with all 10 items revealed misfit in item 10 (evening fatigue), so we removed this item (Table 4). The second iteration displayed overfit in item 2 (exhausted), which was also removed. In the third iteration, all eight items demonstrated acceptable fit to the Rasch model. Then we assessed dimensionality of the remaining eight items by a PCA. The residuals explained by the latent trait was just above 55%; however, the eigenvalue in the 1st component was slightly elevated, justifying further investigation. Residual correlations between items 3 and 4 (mental fatigue), as well as items 5 and 6 (physical fatigue), were above the critical value. This was expected since these item pairs were almost identical and previously flagged from the cognitive interviews. We removed items 4 and 6 and re-ran the analysis. Removing these locally dependent items improved the results indicating unidimensionality (Table 4; Online Resource 5). The remaining six items also demonstrated evidence of local independence, with no positive correlations. The mean person response was about 1 logit higher than the mean item measure. The Wright map (Fig. 2) shows that the subscale works across different levels of fatigue in this sample. In addition, the 6-item subscale demonstrated acceptable KR-20 and person separation (Table 4). Slightly exceeding our criterion, 10 persons (5.9%) demonstrated misfit to the Rasch model. However, no significant differences in the group of misfits compared to the rest of the sample were found in relation to age, gender, or fatigue. Uniform DIF was not detected in relation to gender, but significant DIF was found for item 8 (fatigued around noon) in relation to age, with the age group 61–83 being more likely to agree with this item than the age group 45–60 (p = 0.0064). The final 6-item Characteristics subscale demonstrated evidence of good structural validity and internal consistency. Characteristics subscale raw scores range from six to 30 and correspond to Rasch person measures of -5.96 to 6.37 logits. A score-to-measure table is provided in Online Resource 6.

**Table 4:** Overview of Rasch analysis results at each step in the iterative item removal process

Measurement property	Characteristics subscale		Interference subscale		
Model	Step #1	Step #2	Step #1	Step #2	Step #3
Item goodness-of-fit statistics (Infit MNSQ)					
1st iteration	Item 10 (1.38)		Item 16 (1.41)		
2nd iteration	Item 2 (0.56)		Item 15 (1.48)		
Removed item (reason)	Item 10 (misfit) Item 2 (misfit and flagged)	Item 4 (flagged and locally dependent) Item 6 (flagged and locally dependent)	Item 16 (misfit) Item 15 (misfit)	Item 14 (locally dependent) Item 18 (locally dependent)	Item 27 (flagged) Item 28 (flagged)
Items left for further analysis	8	6	16	14	12
Principal components analysis (PCA) of the residuals:					
Latent trait %	55.4%	57.6%	62.0%	62.9%	62.8%
1st component (eigenvalue)	2.3	1.5	2.5	2.3	2.0
Local independence (Yen’s Q3 residual correlations between items)	Items 5 and 6 (0.55) Items 3 and 4 (0.37)	No positive correlations	Items 12 and 14 (0.37) Items 13 and 14 (0.37) Items 18 and 19 (0.33) Items 17 and 18 (0.31) Items 27 and 28 (0.25) Items 19 and 20 (0.24) Items 25 and 28 (0.24)	Items 11 and 12 (0.25) Items 19 and 20 (0.24) Items 17 and 23 (0.23)	Items 19 and 20 (0.20) Items 11 and 12 (0.20) Items 17 and 23 (0.20) Items 21 and 25 (0.20)
Targeting of persons^a^ Mean person measure (mean model standard error)	1.08 (0.58)	0.95 (0.67)	0.52 (0.42)	0.57 (0.45)	0.55 (0.48)
KR-20 (internal consistency)	0.89	0.86	0.95	0.95	0.94
Person separation index (person reliability)^a^	2.45 (0.86)	2.21 (0.83)	4.11 (0.94)	3.87 (0.94)	3.52 (0.93)
Item separation index (item reliability)	4.11 (0.94)	3.63 (0.93)	5.51 (0.97)	5.87 (0.97)	6.28 (0.98)
Person misfit, *n* (%)	11 (6.5)	10 (5.9)	17 (10)	17 (10)	17 (10)

^a^With extreme and non-extreme persons

#### Interference subscale

**Fig. 3:**
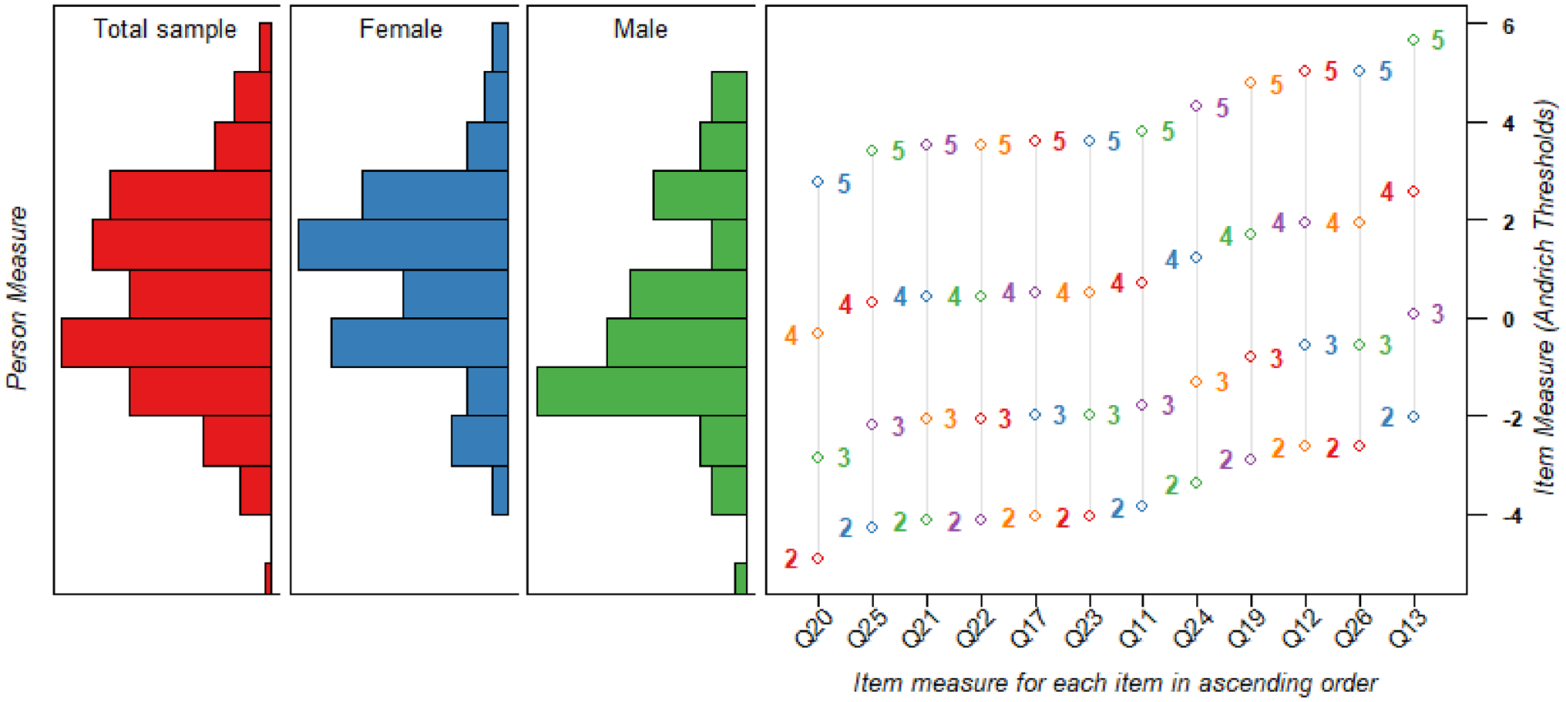
Wright map displaying the 12-item Interference subscale. The left side display individual participants ability measures (based on their mean logits), presented both as a total sample, and separately for females and males. The right side displays the individual items’ difficulty calibrations, including the difficulty of each step calibration (Andrich thresholds). Both the participants’ ability measures and the individual item difficulty calibrations are spaced along the common vertical axis with the logits presented on the right side [35]

Item goodness-of-fit statistics from the first iteration of the 18-item Interference subscale indicated misfit in items 15 (bath/shower) and 16 (dressed/undressed) (Table 4). After removal of these two items, the remaining 16 items demonstrated acceptable fit with infit MNSQ values that met our specified criteria. PCA showed that 62% of the variation was explained by the latent trait; however, as the eigenvalue in the 1st component was greater than 2, and some of the residual item correlations were above 0.3 (indicating locally dependent items), further investigation was justified. Items 14 (gathering thought) and 18 (completing tasks) both demonstrated a higher-than-expected residual correlation with two other items (Table 4); thus, we decided to remove items 14 and 18. We additionally removed items 27 (hobbies) and 28 (pleasant activities), since they were flagged as redundant items from a content validity perspective in the cognitive interviews. In the final subscale, the mean person measure was 0.55 logits higher than the mean item measure, and the Wright map (Fig. 3) shows that the subscale works well across all levels of fatigue in this sample. The final Interference subscale also had a high person separation index and reliability. Fourteen persons (8.28%) demonstrated misfit to the Rasch model on the Interference subscale, which exceeded our criterion (Table 4). However, no statistically significant differences were found in the group of misfitting persons compared to the rest of the sample in relation to age, gender, or fatigue. No significant uniform DIF was found in relation to gender or age groups. Interference subscale raw scores range from 12 to 60 and correspond to Rasch person measures of -7.54 to 8.09 logits. A score-to-measure table is provided in Online Resource 7. In sum, the final 12-item Interference subscale demonstrated good fit to the Rasch model and good structural validity and internal consistency (Table 4; Online Resource 8).

Pearson correlation indicated a strong positive relationship (*r* = 0.77, *p* < 0.001) between each individual’s Rasch measure from the 6-item Characteristic subscale and 12-item Interference subscale.

## Discussion

In this study, we developed and evaluated the Norwegian Fatigue Characteristics and Interference Measure (FCIM), a new 20-item PROM for PSF.

Several previous studies have concluded that fatigue is a multidimensional phenomenon [12, 39]. In this study, based on our conceptual framework and results from the cognitive interviews, the fatigue dimensions of Characteristics and Interference were separated into two subscales. In clinical settings, separating Characteristics and Interference into two subscales is an advantage as the Characteristics subscale can be used in all post-stroke phases, whereas the Interference subscale only can be used after the initial acute phase once the patient has experienced how fatigue has interfered with their life. In addition, differentiating these subscales may also support targeting of different types of interventions. For example, a specific intervention might have no effect on fatigue’s intensity (measured by items in the Characteristics dimension), but could alter fatigue’s interference with the person’s activities. However, the subscales were relatively highly correlated, and future studies with larger and more diverse samples are needed to statistically confirm this two-dimensional structure.

A high-quality PROM needs to be feasible in addition to having evidence of validity and reliability. To avoid respondents losing concentration or becoming fatigued, it is recommended that the instrument not be too extensive or time-consuming [9]. Thus, we aimed for a relatively small number of items in the final FCIM. Three items were removed due to high infit MNSQ values indicating that answers to these items were more unexpected than predicted by the Rasch model. This might indicate that these items capture an additional construct [35]. For example, a possible explanation of misfit in item 10 is that evening fatigue can be commonly experienced even by people with low levels of overall fatigue, as it might reflect normal circadian rhythms of lower energy levels later in the day [40]. Keeping this item could possibly bias the results. A clinical advantage of using a Rasch model to generate individual measures (in logits) is that these measures are based on the individual pattern of responses, not the sum score. Thus, even for respondents with some missing data (e.g., due to fatigue), a Rasch model can generate a comparable measure for use in clinical practice or research (although with a larger individual standard error).

In our previous study, we found that stroke survivors qualitatively described fatigue in a variety of ways [12], a finding also reflected in existing fatigue PROMs using a wide range of different expressions to capture fatigue [6]. Even slight differences in item wording are known to affect responses [41]. To identify the best fatigue wording, we included three item pairs with slightly different wording in the Characteristics subscale. Based on the cognitive interviews, we flagged these items and retained them for the Rasch analysis. Not surprisingly, Rasch analysis indicated local dependence or overfit to the model in these items. It seems likely that the residual correlations between these items indicated duplication (redundancy-dependency) rather than multidimensionality [36]. In retrospect, we could have removed these items after the cognitive interviews, however, it is unlikely that keeping them changed our overall results.

Another limitation of our study was the loss of some separation ability due to the subscales’ item reduction, although the instrument’s structural validity and internal consistency increased, as did its feasibility. Person misfit was slightly higher than expected and should be monitored more closely in larger studies, as a larger sample (> 200) would offer more powerful data on person and context [24]. In addition, we used a convenience sampling method, and despite our sample’s diverse sociodemographic and clinical characteristics, a random-sampling method could yield more robust results.

An advantage of our study is that FCIM is developed based on qualitative data. Our previous review of PROMs used in PSF research showed that existing instruments included items confounded by other post-stroke sequela [6], such as “Do you feel weak?” Including such items could bias the results in a stroke population. Fatigue characteristics and interference are common in other diagnoses, and FCIM has the potential to be used across different patient groups after assessment of its measurement properties.

## Conclusion

In this study, we have developed the FCIM, a patient-reported outcome measure for post-stroke fatigue that includes two subscales measuring fatigue’s Characteristics and Interference, and two pre-stroke fatigue items. This study has shown that the FCIM comprehensively captures the essential experiences of fatigue, and thus, demonstrates evidence of content validity. Using Rasch analysis on the two separate subscales, we removed misfitting and locally dependent items, which resulted in both subscales demonstrating evidence of structural validity and internal consistency. The findings suggest that a scale with relatively few items (from a larger item pool) can be clinically sufficient to generate valid measures of different experiences of fatigue for a vulnerable target group. Further assessment of the FCIM is still necessary before it can be used in clinical practice as well as research. The most important next step is to investigate the FCIM’s ability to detect change over time (i.e., responsiveness) and its relationship to other instruments. Responsiveness is especially important for determining whether the FCIM can be used as an outcome measure for intervention studies. Such studies can also serve as a foundation for using the FCIM to support intervention planning, to minimize fatigue’s severity and interference in daily life for stroke patients. Future studies should also evaluate the FCIM’s measurement properties in other patient populations, as fatigue characteristics and interference are common outcomes of many diseases/disorders. While FCIM is currently only available in Norwegian, we aim to translate and cross-culturally validate the instrument in an English-speaking sample.

### Supplementary Information

Below is the link to the electronic supplementary material.

Supplementary file1 (DOCX 14 kb)

Supplementary file2 (DOCX 19 kb)

Supplementary file3 (DOCX 50 kb)

Supplementary file4 (DOCX 36 kb)

Supplementary file5 (DOCX 12 kb)

Supplementary file6 (DOCX 12 kb)

Supplementary file7 (DOCX 13 kb)

Supplementary file8 (DOCX 12 kb)

## Data Availability

The authors retain copyright for the Norwegian Fatigue Characteristics and Interference Measure (FCIM). For any inquiry regarding the availability, use, translation and scoring of FCIM, contact the first author Ingrid Johansen Skogestad; https://orcid.org/0000-0001-8252-258X.

## References

[1] Paciaroni M, Acciarresi M (2019). Poststroke fatigue. Stroke.

[2] Lanctot KL, Lindsay MP, Smith EE, Sahlas DJ, Foley N, Gubitz G, Austin M, Ball K, Bhogal S, Blake T, Herrmann N, Hogan D, Khan A, Longman S, King A, Leonard C, Shoniker T, Taylor T, Teed M (2020). Canadian stroke best practice recommendations: Mood, cognition and fatigue following stroke, 6th edition update 2019. International Journal of Stroke.

[3] Norwegian Directorate of Health. The Norwegian guideline for stroke treatment and rehabilitation [in Norwegian] 2017. Retrieved from https://www.helsedirektoratet.no/retningslinjer/hjerneslag.

[4] Wu S, Kutlubaev MA, Chun HY, Cowey E, Pollock A, Macleod MR, Dennis M, Keane E, Sharpe M, Mead GE (2015). Interventions for post-stroke fatigue. Cochrane Database of Systematic Reviews.

[5] Kennedy C, Kidd L (2018). Interventions for post-stroke fatigue: A Cochrane review summary. International Journal of Nursing Studies..

[6] Skogestad, I. J., Kirkevold, M., Indredavik, B., Gay, C. L., Lerdal, A., & Group N (2019). Lack of content overlap and essential dimensions—A review of measures used for post-stroke fatigue. Journal of Psychosomatic Research..

[7] Cumming TB, Yeo AB, Marquez J, Churilov L, Annoni J-M, Badaru U, Ghotbi N, Harbison J, Kwakkel G, Lerdal A, Mills R, Naess H, Nyland H, Schmid A, Tang WK, Tseng B, van de Port I, Mead G, English C (2018). Investigating post-stroke fatigue: An individual participant data meta-analysis. Journal of Psychosomatic Research..

[8] Terwee CB, Prinsen CAC, Chiarotto A, Westerman MJ, Patrick DL, Alonso J, Bouter LM, de Vet HCW, Mokkink LB (2018). COSMIN methodology for evaluating the content validity of patient-reported outcome measures: A Delphi study. Quality of Life Research..

[9] de Vet H, Terwee C, Mokkink L, Knol D (2011). Measurement in medicine: A practical guide (practical guides to biostatistics and epidemiology).

[10] U. S. Department of Health Human Services, & F. D. A. (2006). Guidance for industry: Patient-reported outcome measures: use in medical product development to support labeling claims: Draft guidance. *Health and Quality of Life Outcomes*, *4*, 79. 10.1186/1477-7525-4-79 PMC1629006 17034633

[11] Prinsen CAC, Mokkink LB, Bouter LM, Alonso J, Patrick DL, de Vet HCW, Terwee CB (2018). COSMIN guideline for systematic reviews of patient-reported outcome measures. Quality of Life Research.

[12] Skogestad IJ, Kirkevold M, Larsson P, Borge CR, Indredavik B, Gay CL, Lerdal A (2021). Post-stroke fatigue: An exploratory study with patients and health professionals to develop a patient-reported outcome measure. Journal of Patient Reported Outcomes.

[13] Nadarajah M, Goh HT (2015). Post-stroke fatigue: A review on prevalence, correlates, measurement, and management. Topics in Stroke Rehabilitation.

[14] Lerdal A, Kottorp A (2011). Psychometric properties of the Fatigue Severity Scale-Rasch analyses of individual responses in a Norwegian stroke cohort. International Journal of Nursing Studies.

[15] Krupp LB, LaRocca NG, Muir-Nash J, Steinberg AD (1989). The fatigue severity scale. Application to patients with multiple sclerosis and systemic lupus erythematosus. Archives of Neurology..

[16] Elbers RG, Rietberg MB, van Wegen EE, Verhoef J, Kramer SF, Terwee CB, Kwakkel G (2012). Self-report fatigue questionnaires in multiple sclerosis, Parkinson's disease and stroke: A systematic review of measurement properties. Quality of Life Research.

[17] Oosterveld P, Vorst HCM, Smits N (2019). Methods for questionnaire design: A taxonomy linking procedures to test goals. Quality of Life Research.

[18] Mokkink LB, Terwee CB, Patrick DL, Alonso J, Stratford PW, Knol DL, Bouter LM, de Vet HC (2010). The COSMIN study reached international consensus on taxonomy, terminology, and definitions of measurement properties for health-related patient-reported outcomes. Journal of Clinical Epidemiology.

[19] Andrich, D., & Marais I. (2019). A course in Rasch measurement theory. *D Andrich y I Marais (Coords), Measuring in the Educational, Social and Health Sciences,**41*, 53.

[20] Edelen MO, Reeve BB (2007). Applying item response theory (IRT) modeling to questionnaire development, evaluation, and refinement. Quality of Life Research.

[21] Mead G, Lynch J, Greig C, Young A, Lewis S, Sharpe M (2007). Evaluation of fatigue scales in stroke patients. Stroke.

[22] Tong A, Sainsbury P, Craig J (2007). Consolidated criteria for reporting qualitative research (COREQ): A 32-item checklist for interviews and focus groups. International Journal for Quality in Health Care.

[23] Mallinson T, Kozlowski AJ, Johnston MV, Weaver J, Terhorst L, Grampurohit N, Juengst S, Ehrlich-Jones L, Heinemann AW, Melvin J, Sood P, Van de Winckel A (2022). Rasch reporting guideline for rehabilitation research (RULER): The RULER statement. Archives of Physical Medicine and Rehabilitation.

[24] Mokkink LB, Prinsen CA, Patrick DL, Alonso J, Bouter LM, de Vet HC, Terwee CB (2019). COSMIN study design checklist for patient-reported outcome measurement instruments.

[25] Patrick DL, Burke LB, Gwaltney CJ, Leidy NK, Martin ML, Molsen E, Ring L (2011). Content validity–establishing and reporting the evidence in newly developed patient-reported outcomes (PRO) instruments for medical product evaluation: ISPOR PRO good research practices task force report: Part 1–eliciting concepts for a new PRO instrument. Value in Health..

[26] Patrick DL, Burke LB, Gwaltney CJ, Leidy NK, Martin ML, Molsen E, Ring L (2011). Content validity–establishing and reporting the evidence in newly developed patient-reported outcomes (PRO) instruments for medical product evaluation: ISPOR PRO Good Research Practices Task Force report: Part 2–assessing respondent understanding. Value in Health..

[27] Gagnier JJ, Lai J, Mokkink LB, Terwee CB (2021). COSMIN reporting guideline for studies on measurement properties of patient-reported outcome measures. Quality of Life Research.

[28] Tourangeau R, Jabine TB, Tanur JM, Tourangeau R (1984). Cognitive science and survey methods. Cognitive aspects of survey design: Building a bridge between disciplines.

[29] Hak T, Veer KVD, Jansen H (2008). The three-step test-interview (TSTI): An observation-based method for pretesting self-completion questionnaires. Survey Research Methods.

[30] Elo S, Kyngas H (2008). The qualitative content analysis process. Journal of Advanced Nursing.

[31] Pallant JF, Tennant A (2007). An introduction to the Rasch measurement model: An example using the Hospital Anxiety and Depression Scale (HADS). British Journal of Clinical Psychology.

[32] Linacre JM (2002). Optimizing rating scale category effectiveness. Journal of Applied Measurement.

[33] Bond TG, Fox CM (2013). Applying the Rasch model: Fundamental measurement in the human sciences.

[34] Wright BD, Masters GN (1982). Rating scale analysis.

[35] Linacre, J. M. (2006). A user's guide to Winsteps Ministeps Rasch-model computer programs. Program Manual 5222022.

[36] Christensen KB, Makransky G, Horton M (2017). Critical values for Yen's Q3: Identification of local dependence in the Rasch model using residual correlations. Applied Psychological Measurement..

[37] Tennant A, Conaghan PG (2007). The Rasch measurement model in rheumatology: What is it and why use it? When should it be applied, and what should one look for in a Rasch paper?. Arthritis and Rheumatology..

[38] Lerdal A (2013). Curvilinear relationship between age and post-stroke fatigue among patients in the acute phase following first-ever stroke. International Journal of Physical Medicine & Rehabilitation.

[39] Lerdal A, Bakken LN, Kouwenhoven SE, Pedersen G, Kirkevold M, Finset A, Kim HS (2009). Poststroke fatigue–a review. Journal of Pain and Symptom Management..

[40] Valdez P (2019). Circadian rhythms in attention. The Yale Journal of Biology and Medicine.

[41] Willis GB (2004). Cognitive interviewing: A tool for improving questionnaire design.

[42] Smith AB, Rush R, Fallowfield LJ, Velikova G, Sharpe M (2008). Rasch fit statistics and sample size considerations for polytomous data. BMC Medical Research Methodology.

[43] Mokkink, L. B., Prinsen, C. A., Patrick, D., Alonso, J., Bouter, L. M., De Vet, H. C., & Terwee, C. B. (2018). COSMIN methodology for systematic reviews of Patient‐Reported Outcome Measures (PROMs) User Manual. 10.1007/s11136-018-1798-3 PMC5891568 29435801

[44] Benjamini Y, Hochberg Y (1995). Controlling the false discovery rate: A practical and powerful approach to multiple testing. Journal of the Royal Statistical Society Series B (Methodological)..

[45] Lerdal A, Bakken LN, Rasmussen EF, Beiermann C, Ryen S, Pynten S, Drefvelin AS, Dahl AM, Rognstad G, Finset A, Lee KA, Kim HS (2011). Physical impairment, depressive symptoms and pre-stroke fatigue are related to fatigue in the acute phase after stroke. Disability and Rehabilitation.

