# Supplementary material for: Development and evaluation of the Norwegian Fatigue Characteristics and Interference Measure (FCIM) for stroke survivors: cognitive interviews and Rasch analysis
Source: Qual Life Res. 2023 Jul 19;32(12):3389–401. doi: 10.1007/s11136-023-03477-z (PMC10624711; doi:10.1007/s11136-023-03477-z)
Supplement: Supplementary file 1 — Supplementary file1 (DOCX 14 kb) [file 11136_2023_3477_MOESM1_ESM.docx]

**Online resource 1 - Cognitive Interview Guide**

| **Cognitive Interview Guide** |
| --- |
| **Introduction:** We have developed a new instrument to assess fatigue in stroke patients. The aim of this interview is to test our new instrument and find out whether you find the items difficult to answer, if you understand what we are asking, if we lack response options etc. We are not so interested in what your actual answer is, but rather how you experience the process of answering.  This interview will have three steps. First, I want you to answer the instrument, and concurrently think aloud while you are answering. In this step, I will only observe and cannot answer any questions that you might have. In the next step, I will follow up on the observations I made in the first step, and lastly, we will talk more freely about the instrument and your experiences. |
| **Step 1 – Observation**  Observe the respondent’s behavior and gather “think aloud” data. Look specifically for:  -Questions that are being skipped  -Correction of the chosen response category  -Hesitation  -Stress/insecurity |
| **Step 2 – Follow up probing**  Ask questions about the observations made in step 1. For example:  -You stopped by this question, what did you think then?  -Did I hear you saying…? |
| **Step 3 – De-briefing about experiences and opinions**  Ask questions about the respondent’s experiences of and opinions about specific items and the instrument as a whole. For example:  -How did you understand the introduction to the instrument?  -How did you comprehend this term in this question?  -Can you tell me why you answered alternative x on this question?  -Do you have any suggestions to how this could have been formulated differently?  -You had problems answering question x, can you tell me about how your situation is related to this question?  -How do you understand the answer alternatives in this question?  -Did any of the questions seem excessive/irrelevant?  -Were there any questions or themes that you thought were missing?  -Overall, what did you think of the instrument as a whole measuring post-stroke fatigue? |
