# Supplementary material for: Development and evaluation of the Norwegian Fatigue Characteristics and Interference Measure (FCIM) for stroke survivors: cognitive interviews and Rasch analysis
Source: Qual Life Res. 2023 Jul 19;32(12):3389–401. doi: 10.1007/s11136-023-03477-z (PMC10624711; doi:10.1007/s11136-023-03477-z)
Supplement: Supplementary file 2 — Supplementary file2 (DOCX 19 kb) [file 11136_2023_3477_MOESM2_ESM.docx]

**Online Resource 2 – Item-tracking matrix of FSCIM Version 1.0**

| **Introduction** | | **Analytical summary** | **Example quotes** | **Changes made (after 10 or 15 interviews)** | **Results** |
| --- | --- | --- | --- | --- | --- |
| Post-stroke fatigue definition, long version | | Problems with reading and understanding the long definition |  | Simplified the definition after 10 interviews | No problems in the last 5 interviews |
| **Severity subscale** | | | | |  |
| Item | Short item name | Analytical summary | Example quotes | Changes made (after 10 or 15 interviews) | Results |
| 1 | Fatigued |  |  |  |  |
| 2 | Exhausted | Several interpreted this item as the feeling you get after a hard physical exercise out or intensive work | *“Exhausted, that is what you are after a long day at work.” Participant 10* |  | **Flagged item**  **(removed after Rasch)** |
| 3 | Mentally fatigued |  |  |  |  |
| 4 | Tired in your head | This was comprehended as being almost the same as item 3, but as a slightly wider concept, e.g. also including head pain and worrying for the future | *“I feel that tired in your head, that is similar to mentally fatigue […] but tired in your head can also be headache, or that your feelings are gathering up…” Participant 8* |  | **Flagged item (removed after Rasch)** |
| 5 | Physically fatigued |  |  |  |  |
| 6 | Tired in your body | This was comprehended as being almost the same as item 5, but as also including body pain and normal tiredness after exercise. | “Being tired in your body… that could also be after physical exercise and that’s a good feeling.” Participant 5  “I think that is almost the same, physically fatigued and tired in your body.” Participant 15 |  | **Flagged item**  **(removed after Rasch)** |
| 7 | Morning fatigue |  |  |  |  |
| 8 | Fatigued around noon |  |  |  |  |
| 9 | Afternoon fatigue |  |  |  |  |
| 10 | Evening fatigue |  |  |  | **(removed after Rasch)** |
| *Interference subscale* | | | | |  |
| 11 | Concentrating | Initial wording: “How often did you have problems concentrating due to fatigue?” This wording were comprehended as two questions in one: one about concentration and the other about fatigue. | *“I have problems with my concentration, but that has not anything to do with my fatigue… other than that it gets worse when I am fatigued.” Participant 3* | Changed after 10 interviews to: “How often were you so fatigued that you had problems concentrating? | No problems in the last 5 interviews. |
| 12 | Making decisions | Initial wording: “How often did you have problems making decisions due to fatigue?” This wording was comprehended as two questions in one: one about making decisions and the other about fatigue. |  | Changed after 10 interviews to: “How often were you so fatigued that you had problems making decisions. | No problems in the last 5 interviews. |
| 13 | Following a conversation | Initial wording: “How often did you have problems following a conversation due to fatigue?” This wording was comprehended as two questions in one: one about following a conversation and the other about fatigue. | *“If I am out at social gatherings, then I fall out [of the conversation], because it gets too much different voices and sounds…” Participant 1* | Changed after 10 interviews to: “How often were you so fatigued that you had problems following a conversation. | No problems in the last 5 interviews. |
| 14 | Gathering thoughts | A few commented that this item was very similar to item 11, concentrating.  Initial wording: “How often did you have problems gathering your thoughts due to fatigue?” This wording was comprehended as two questions in one: one about gathering thoughts and the other about fatigue. |  | Changed after 10 interviews to: “How often were you so fatigued that you had problems gathering your thoughts. | **No problems in the last 5 interviews. (Item 14 removed after Rasch)** |
| 15 | Bath or shower |  |  |  | **(removed after Rasch)** |
| 16 | Dress/undress |  |  |  | **(removed after Rasch)** |
| 17 | Starting activities | We detected problems with item 17-20. These items were interpreted differently than our intention. We changed individual wording of these items as described below. We also changed the ordering of items, and put these four items together in a more logical sequence. After completing these changes, we did not experience any problems in the last 5 interviews.    Some respondents had problems with comprehension and judgement because of the overall wording of the item. Our aim was to investigate if they had problems **starting** things in general, but the respondents rather emphasized problems with daily tasks. Initial wording: “How often were you unable to get started with daily tasks due to fatigue?” | *“Daily tasks… what do you mean by daily tasks?” Respondent 4* | Changed the wording after 10 interviews to: “How often did you have problems starting your tasks/activities because of fatigue?” | No problems in the last 5 interviews. |
| 18 | Completing activities | We detected problems with comprehension of this item. It was interpreted as the focus was on household chores, and many did not have any specific chores. Our aim was to investigate if they had problems **completing** things in general, and our initial wording was not reflecting this aim.  Initial wording: “How often did you have problems completing your tasks at home due to fatigue” | *“Several things I leave to my husband now, such as vacuuming.” Respondent 6* | Changed the wording after 10 interviews to: “How often did you have problems completing your tasks/activities because of fatigue?” | No problems in the last 5 interviews.  **removed after Rasch)** |
| 19 | Giving up on activities | We detected problems with comprehension, because of the overall wording of the item. Initial wording: “How often did you have to stop an activity due to fatigue?” |  | Changed the wording after 10 interviews to: “How often did you have to give up on your tasks/activities because of fatigue?” | No problems in the last 5 interviews. |
| 20 | Taken more time | Judgement problems. Some choose to take more time as a coping mechanism. Initial wording: “How often have tasks taken longer time because of fatigue?” |  | Changed the wording after 10 interviews to: “How often have tasks/activities taken more time because of fatigue?” | No problems in the last 5 interviews. |
| 21 | Activities outside home |  |  |  |  |
| 22 | Planning |  |  |  |  |
| 23 | Not done anything |  |  |  |  |
| 24 | Physical activity |  |  |  |  |
| 25 | Social activity |  |  |  |  |
| 26 | Family |  |  |  |  |
| 27 | Hobbies | Comprehension and retrieval problems. Some respondents had no hobbies. Overlaps with other items. We decided to flag this item and investigate how it performed in the Rasch analysis. | *“Hobbies? I think this item is very similar to social activities. Is it common for adults to have hobbies other than physical exercise? Participant 10*  *“This is difficult to answer since I don’t have any hobbies. But I ended up answering never” Participant 11* |  | **Flagged item**  **(removed after Rasch)** |
| 28 | Pleasant activities | Comprehension and judgement problems. Overlaps with other items. We decided to keep this item for further Rasch analysis. | *“…social activities, there I responded often, and then pleasant activities… These items are overlapping I think…” Participant 5* |  | **Flagged item**  **(removed after Rasch)** |
| 29 | Rehabilitation | Initial wording was: “If you have rehabilitation therapy, how often did you avoid planned sessions due to fatigue?”  Participants reported problems with comprehension, retrieval and judgement. Several did not understand what we meant with “rehabilitation therapy” and we changed the wording after 10 interviews. These changes made the item similar to item 24 and was then comprehended as general physical activity. Some participants were not engaged with rehabilitation activities. Considering these overall problems, we decided to remove this item. | *“I don’t know what you mean by rehabilitation activities? Do you mean physical activity?” Participant 6.* | “If you are doing retraining after your stroke, how often were you so fatigued that you avoided exercises? | **Removed after 15 interviews due to problems and lack of relevance** |
| 30 | Paid work | Wording “If you are working, how often were you less effective at work due to fatigue?” This item had the same response alternatives as the other questions and some respondents answered this question even if they were on full disability leave. If included, this item should have a “not relevant” category since it was not relevant to the respondents who did not work. We decided that we only wanted to include items that were relevant for all participants, and that item 21 (activities outside home) could partly enable participants to report interference with work in that item. | *“If you are at work… I am not at work… What shall I answer on this item since I am not working?” Participant 13* |  | **Removed after 15 interviews due to lack of relevance** |
| 31 | Pre-stroke fatigue |  |  |  |  |
| 32 | Pre-stroke fatigue | Problems with two overlapping response categories. Initial wording was “1 month” and “1-6 months”. This was changed to “1 month or less” and “2-6 months” |  | Changed response categories after 10 interviews. | No problems in the last 5 interviews. |
| *Overall judgement of completeness* | | Changed the order of almost all items in the interference subscale after 10 interviews. The pre-stroke items were moved from the start to the end of the instrument. The participants did not identify any missing items or topics that were relevant to the assessment of post-stroke fatigue. | | |  |

Notes: Blank cells indicate that no problems were identified and/or no changes were made. Flagged items had problems identified in the cognitive interviews but were retained for further evaluation in Rasch analysis. All items flagged in the cognitive interviews were subsequently removed based on the Rasch analysis.
