# Supplementary material for: Development and evaluation of the Norwegian Fatigue Characteristics and Interference Measure (FCIM) for stroke survivors: cognitive interviews and Rasch analysis
Source: Qual Life Res. 2023 Jul 19;32(12):3389–401. doi: 10.1007/s11136-023-03477-z (PMC10624711; doi:10.1007/s11136-023-03477-z)

**Online resource 3 – Andrich thresholds with corresponding standard errors of the 6-item characteristics scale**


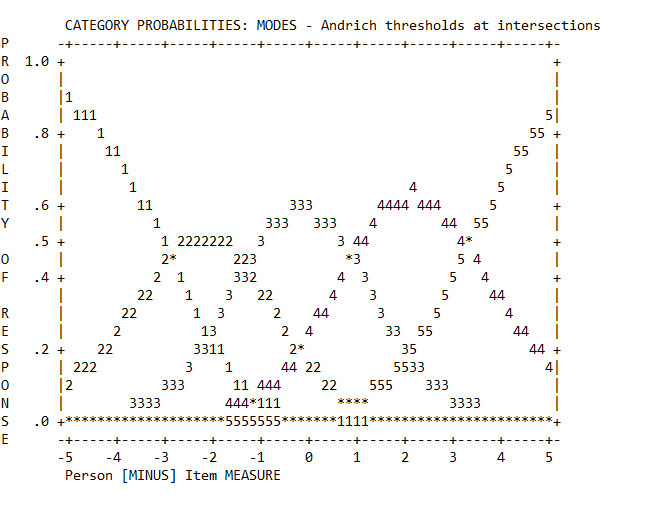


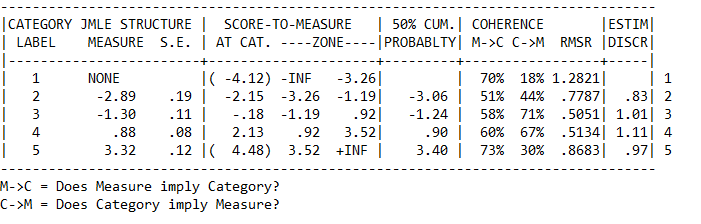

Supplement: Supplementary file 3 — Supplementary file3 (DOCX 50 kb) [file 11136_2023_3477_MOESM3_ESM.docx]
