# Supplementary material for: Development and evaluation of the Norwegian Fatigue Characteristics and Interference Measure (FCIM) for stroke survivors: cognitive interviews and Rasch analysis
Source: Qual Life Res. 2023 Jul 19;32(12):3389–401. doi: 10.1007/s11136-023-03477-z (PMC10624711; doi:10.1007/s11136-023-03477-z)
Supplement: Supplementary file 5 — Supplementary file5 (DOCX 12 kb) [file 11136_2023_3477_MOESM5_ESM.docx]

**Online resource 5 – The 6 item characteristics subscale showing item calibrations and fit statistics reported in hierarchical order, difficulty from most to least.**

| Items | Measure (logits) | Std. error | Infit MnSq | Infit zstd | Outfit MnSq | Outfit zstd |
| --- | --- | --- | --- | --- | --- | --- |
| 8 | 0.60 | 0.12 | 0.88 | -1.13 | 0.88 | -1.08 |
| 7 | 0.46 | 0.12 | 1.15 | 1.32 | 1.16 | 1.44 |
| 5 | 0.24 | 0.12 | 1.20 | 1.73 | 1.22 | 1.89 |
| 3 | -0.23 | 0.12 | 1.11 | 0.99 | 1.08 | 0.76 |
| 1 | -0.36 | 0.12 | 0.71 | -2.93 | 0.70 | -3.07 |
| 9 | -0.70 | 0.12 | 0.90 | -0.95 | 0.93 | -0.62 |

*Std., standard; MnSq, mean square; zstd, Z standard.
