# Supplementary material for: Development and evaluation of the Norwegian Fatigue Characteristics and Interference Measure (FCIM) for stroke survivors: cognitive interviews and Rasch analysis
Source: Qual Life Res. 2023 Jul 19;32(12):3389–401. doi: 10.1007/s11136-023-03477-z (PMC10624711; doi:10.1007/s11136-023-03477-z)
Supplement: Supplementary file 6 — Supplementary file6 (DOCX 12 kb) [file 11136_2023_3477_MOESM6_ESM.docx]

**Online resource 6 - Raw score to Rasch logits conversion table of the 6-item characteristics subscale of FCIM**

For your data (6-item characteristics subscale), it would be S = 100/(5.96 + 6.37) = 8.1103 M= 0 - (-5.96*8.1103) = 48.33739 formula in column converted logits to 0-100 Y = 48.33739 + (8.1103 * B2) --> cell B2 and following would be the column with the logits.

| FCIM characteristics raw score | FCIM characteristics Rasch logits | FCIM characteristics percentages |
| --- | --- | --- |
| 6 | -5.96 | 0 |
| 7 | -4.67 | 0 |
| 8 | -3.85 | 1 |
| 9 | -3.31 | 1 |
| 10 | -2.88 | 1 |
| 11 | -2.50 | 2 |
| 12 | -2.16 | 4 |
| 13 | -1.83 | 7 |
| 14 | -1.51 | 9 |
| 15 | -1.18 | 12 |
| 16 | -0.86 | 16 |
| 17 | -0.52 | 21 |
| 18 | -0.17 | 27 |
| 19 | 0.19 | 35 |
| 20 | 0.55 | 43 |
| 21 | 0.93 | 53 |
| 22 | 1.32 | 62 |
| 23 | 1.72 | 68 |
| 24 | 2.14 | 74 |
| 25 | 2.58 | 83 |
| 26 | 3.04 | 89 |
| 27 | 3.55 | 93 |
| 28 | 4.16 | 95 |
| 29 | 5.04 | 96 |
| 30 | 6.37 | 99 |
