# Supplementary material for: Development and evaluation of the Norwegian Fatigue Characteristics and Interference Measure (FCIM) for stroke survivors: cognitive interviews and Rasch analysis
Source: Qual Life Res. 2023 Jul 19;32(12):3389–401. doi: 10.1007/s11136-023-03477-z (PMC10624711; doi:10.1007/s11136-023-03477-z)
Supplement: Supplementary file 7 — Supplementary file7 (DOCX 13 kb) [file 11136_2023_3477_MOESM7_ESM.docx]

**Online resource 7 - Raw score to Rasch logits conversion table of the 12-item interference subscale of FCIM**

| FCIM interference raw score | FCIM interference Rasch logits | FCIM interference percentages |
| --- | --- | --- |
| 12 | -7.54 | 0 |
| 13 | -6.27 | 0 |
| 14 | -5.48 | 0 |
| 15 | -4.97 | 1 |
| 16 | -4.58 | 1 |
| 17 | -4.26 | 1 |
| 18 | -3.97 | 1 |
| 19 | -3.71 | 1 |
| 20 | -3.47 | 1 |
| 21 | -3.24 | 2 |
| 22 | -3.02 | 3 |
| 23 | -2.81 | 4 |
| 24 | -2.61 | 6 |
| 25 | -2.40 | 7 |
| 26 | -2.21 | 8 |
| 27 | -2.01 | 9 |
| 28 | -1.81 | 11 |
| 29 | -1.62 | 12 |
| 30 | -1.42 | 15 |
| 31 | -1.22 | 20 |
| 32 | -1.03 | 22 |
| 33 | -0.82 | 27 |
| 34 | -0.62 | 31 |
| 35 | -0.42 | 34 |
| 36 | -0.21 | 37 |
| 37 | 0.00 | 41 |
| 38 | 0.21 | 45 |
| 39 | 0.43 | 49 |
| 40 | 0.65 | 52 |
| 41 | 0.87 | 55 |
| 42 | 1.10 | 59 |
| 43 | 1.34 | 64 |
| 44 | 1.57 | 68 |
| 45 | 1.82 | 72 |
| 46 | 2.07 | 78 |
| 47 | 2.32 | 83 |
| 48 | 2.58 | 87 |
| 49 | 2.84 | 89 |
| 50 | 3.11 | 91 |
| 51 | 3.38 | 92 |
| 52 | 3.66 | 93 |
| 53 | 3.96 | 95 |
| 54 | 4.27 | 96 |
| 55 | 4.60 | 97 |
| 56 | 4.97 | 99 |
| 57 | 5.40 | 99 |
| 58 | 5.95 | 99 |
| 59 | 6.79 | 100 |
| 60 | 8.09 | 100 |
