# Supplementary material for: Development and evaluation of the Norwegian Fatigue Characteristics and Interference Measure (FCIM) for stroke survivors: cognitive interviews and Rasch analysis
Source: Qual Life Res. 2023 Jul 19;32(12):3389–401. doi: 10.1007/s11136-023-03477-z (PMC10624711; doi:10.1007/s11136-023-03477-z)
Supplement: Supplementary file 8 — Supplementary file8 (DOCX 12 kb) [file 11136_2023_3477_MOESM8_ESM.docx]

**Online resource 8 - The 12-item interference subscale showing item calibrations and fit statistics reported in hierarchical order, difficulty from most to least.**

| Items | Measure (logits) | Std. error | Infit MnSq | Infit zstd | Outfit MnSq | Outfit zstd |
| --- | --- | --- | --- | --- | --- | --- |
| 13 | 1.58 | 0.12 | 1.06 | 0.57 | 1.09 | 0.79 |
| 26 | 0.95 | 0.12 | 0.91 | -0.82 | 0.90 | -0.90 |
| 12 | 0.95 | 0.12 | 1.11 | 1.02 | 1.14 | 1.30 |
| 19 | 0.69 | 0.12 | 1.03 | 0.35 | 1.03 | 0.36 |
| 24 | 0.20 | 0.13 | 1.11 | 1.05 | 1.12 | 1.09 |
| 11 | -0.28 | 0.13 | 0.84 | -1.50 | 0.82 | -1.70 |
| 23 | -0.48 | 0.13 | 0.98 | -0.18 | 0.99 | -0.03 |
| 17 | -0.48 | 0.13 | 0.93 | -0.64 | 0.90 | -0.91 |
| 22 | -0.56 | 0.13 | 1.18 | 1.57 | 1.17 | 1.52 |
| 21 | -0.56 | 0.13 | 1.02 | 0.24 | 1.00 | 0.04 |
| 25 | -0.69 | 0.13 | 0.98 | -0.15 | 0.94 | -0.52 |
| 20 | -1.33 | 0.13 | 0.85 | -1.40 | 0.82 | -1.61 |

*Std., standard; MnSq, mean square; zstd, Z standard.
